# The Effect of Whole Body Vibration Exposure on Muscle Function in Children With Cystic Fibrosis: A Pilot Efficacy Trial

**DOI:** 10.4021/jocmr1137w

**Published:** 2013-04-23

**Authors:** Kaitlin O’Keefe, Rhonda Orr, Peite Huang, Hiran Selvadurai, Peter Cooper, Craig Frank Munns, Maria A Fiatarone Singh

**Affiliations:** a Exercise, Health and Performance, Faculty Research Group, Faculty of Health Sciences, University of Sydney, Australia; b Department of Respiratory Medicine, The Children’s Hospital at Westmead Westmead, Sydney 2145, New South Wales, Australia; c Sydney Medical School, University of Sydney, Australia; d Department of Endocrinology and Diabetes, The Children’s Hospital at Westmead, Sydney 2145, New South Wales, Australia; e Hebrew Senior Life and Jean Mayer USDA Human Nutrition Centre on Aging at Tufts University, Boston, MA, USA

**Keywords:** Cystic Fibrosis, Children, Vibration, Muscle function, Muscle power

## Abstract

**Background:**

To examine the effects of whole body vibration (WBV) exposure on muscle function in children with Cystic Fibrosis (CF). Non-randomised controlled cross-over trial.

**Methods:**

The setting was home-based WBV exposure. The participants were children (8 - 15 years) with CF (n = 7). Intervention: participants served as their own controls for the first four weeks (usual care), then underwent four weeks of parentally-supervised home-based WBV exposure followed by four weeks washout (usual care). The WBV exposure consisted of 20 - 30 minutes of intermittent (1 min vibration:1 min rest) exposure on a Galileo platform (20 - 22Hz, 1 mm amplitude) 3 days/week. The primary outcome measures of absolute and relative lower body (leg extension (LE), leg press (LP)), upper body (chess press (CP)) strength and power, and power were measured at baseline, and weeks 4, 8 and 12. Secondary exploratory outcomes were cardiorespiratory fitness, pulmonary function and health-related quality of life.

**Results:**

Six participants completed the training without adverse events. Muscle function changes following WBV exposure were not statistically significant. However, moderate-to-large relative effect sizes (ES) favouring WBV were evident for leg extension strength (ES = 0.66 (-0.50, 1.82)), LP relative strength (ES = 0.92 (-0.27, 2.11)), leg press peak power (ES = 0.78 (-0.50, 2.07)) and CMJ height (ES = 0.60 (-0.56 to 1.76)).

**Conclusions:**

The results from this first controlled trial indicate that WBV may be a potentially effective exercise modality to safely increase leg strength and explosive power in children with CF. Potentially clinically relevant changes support continued investigation of the efficacy, mechanism and feasibility of this intervention in future large-scale studies.

## Introduction

Cystic fibrosis (CF) is an autosomal recessive disease [1]. Pubescent children with CF are significantly less active than healthy controls [2, 3]. With the progression of this multi-system disease, many patients experience exercise intolerance [4], and less-fit patients are reported to have a poorer prognosis than their aerobically-fit counterparts [5]. Currently, exercise is advocated as a critical part of the care-plan for patients with CF, due to its benefits to aerobic [6-8] and anaerobic fitness [9], respiratory function [10, 11], peripheral muscle strength [1, 6, 7, 10] and health-related quality of life (HRQOL) [9, 12].

Patients with CF have reduced muscle strength when compared to healthy age-matched individuals [13, 14], however there is little consensus as to the aetiology of this muscle weakness [15]. Some studies suggest impairments in the muscle quality and force-generating capacity [16-18], whilst others report that smaller peripheral muscle mass is responsible for the decreased strength [19-21]. Physical inactivity [22, 23], poor nutritional status [15, 24], corticosteroid therapy [15, 25], hypoinsulinaemia due to pancreatic dysfunction [26, 27], and increased resting energy expenditure [28, 29] may all contribute. It is most likely, however, that the primary determinants of reduced fat-free mass are the inflammatory and catabolic responses to chronic lung disease and infection and corticosteroid treatment [22, 30]. As CF life expectancy has increased to an average of 37 years [31], sarcopenia, and CF-related diabetes mellitus and osteoporosis are now emerging in this population, making it even more pertinent that interventions can both treat and prevent these conditions.

Recently, whole body vibration (WBV) has received much attention for its purported ability to improve bone mineral density (BMD) [32], flexibility [33, 34], balance and mobility [35, 36], aerobic capacity [37] and notably, muscle function [38, 39]. WBV has been theorised to act on muscle function in part via the stimulation of muscle spindles, leading to the excitation of alpha motor neurons, which contract the motor units. A tonic vibration reflex, or tonic contraction of the muscle results [40]. As acute exacerbations of CF contribute to fatigue and may preclude participation in traditional modalities of exercise, one potential utility of this modality of training is its non-exertional nature. It could theoretically be a useful alternative/adjunct to traditional resistance or aerobic training exercises, as it can be performed by those unable to exercise, and even continued during periods of severe dyspnoea, illness and hospitalisation for treatment of CF and its co-morbidities.

Recently, two uncontrolled trials have investigated home-based WBV exposure with concomitant muscle strengthening exercise in adults with CF [41, 42]. In the first [42], six months of continuous WBV exposure (6 - 12 minutes; 12 - 26 Hz), five days/week in eleven adults aged 29 - 38 years resulted in small non-significant improvements in muscle power 4.7% (range -16.4% to +74.5%) and velocity 6.6% (range -0.9% to +48.3%), as assessed by one- and two-legged jumps on a Leonardo (Novotec Medical, Pforzheim, Germany) platform. By contrast, in the second study, three months of intermittent WBV exposure (18 minutes) five days/week in 10 adults (three males; seven females) aged 24 - 47 years significantly improved in lower-extremity muscle force and power [41]. Interpretation of these findings is limited due to the uncontrolled study designs, heterogeneous results, and most importantly, the use of a combined intervention of resistance training plus WBV, precluding the attribution of benefits to WBV itself.

Thus, our purpose was to conduct the first controlled efficacy trial of isolated WBV exposure in children with CF. The specific aims of this controlled cross-over trial were to: (1) assess the effect of one month of WBV exposure on muscle strength and power, maximal aerobic capacity, pulmonary function and HRQOL in children with CF; and (2) determine any residual effect of WBV exposure after a washout period; calculate effect sizes and sample size determinations for a full-scale trial.

Our primary hypothesis was that 4 weeks of WBV exposure (3 times/week) would increase muscle function relative to the preceding usual care control period in children with CF. Our secondary hypothesis was that any improvements in musculoskeletal outcomes would not be present at the end of a 4-week washout period. Additional secondary exploratory hypotheses were that WBV exposure would improve cardio-respiratory endurance performance, pulmonary function, and HRQOL.

## Methods

### Participants

Participants were recruited from the CF Clinic at The Children’s Hospital at Westmead (CHW). Inclusionary criteria proven CF and 8 - 18 years of age. Exclusionary criteria were contraindications to WBV [43], severe CF (forced expiratory volume in 1 minute (FEV_1_ < 40% predicted)), inability to stand unaided for 30 minutes, long bone or vertebral fracture in the past six months, past/present history of osteoarthritis, presence of CF-liver disease with portal hypertension, neuropathy or myopathy and/or vitamin D deficiency (25-hydroxyvitamin D < 39 nmol/L) in the past three months. The study was approved by CHW and The University of Sydney Human Research Ethics Committees (July 2008) and registered with the Australian New Zealand Clinical Trials Registry (number: ACTRN12609000090213). Written informed consent was obtained from all participants and parents.

### Study design

**Figure 1:**
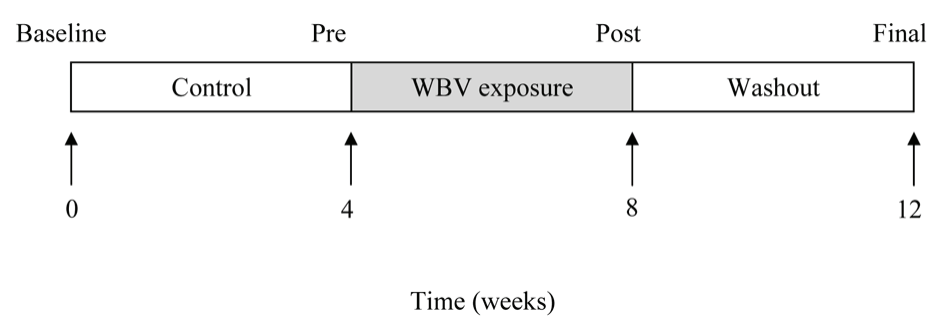
Study Design. The arrows indicate 4 test occasions over the twelve weeks. Baseline values were obtained at week 0, at end of Control period testing at week 4, at end of Vibration exposure period at week 8, and at end of Washout period between week 8 and at week 12. The black rectangle indicates the training weeks, and the white rectangle shows when there was no applied intervention. Usual care was given throughout the twelve weeks. In the Control and Washout periods, participants continued usual physical activity.

This controlled cross-over trial involved four weeks each of control, WBV exposure and washout period. Randomisation of control and exposure periods was not possible due to unknown duration of WBV effect, if any. Tests were conducted at four time points: baseline, week 4 (pre), week 8 (post) and week 12 (final) as shown in Figure 1. Following the training month, testing took place 48 - 72 hours after the last WBV session to avoid acute effects of vibration exposure [44, 45]. Participants were informed not to change levels of physical activity during the study. Usual medical care was continued and monitored but not controlled by study investigators.

### Measurements

Testing took place at CHW and The University of Sydney. The order of testing was standardised. Testing at The University of Sydney was performed by one researcher. Pulmonary function testing took place at CHW by one of two researchers.

### Primary outcomes

#### Muscle function (strength and power)

Strength was assessed on Keiser pneumatic-resistance training equipment^a^ using one repetition maximum (1RM) in three bilateral exercises: knee extension, chest press and horizontal leg press [46]. Power was measured at 60% current 1RM, as peak power has been observed between 50-75% 1RM [47]. The highest of three maximal explosive efforts were performed separated by 1-minute rest was used in analyses.

#### Counter-movement jump (CMJ) performance

Participants performed three maximal CMJs on a force platform^b^. The vertical signal from the platform was sampled at 1,000 Hz and recorded by computer. The highest jump height and the subsequent power and ground reaction forces (GRFs) were recorded and analysed (ICC, test-retest reliability in untrained females, r = 0.99) [48].

### Secondary outcomes and descriptive characteristics

#### Anthropometrics

Height^d^ and weight^e^ were measured, and body mass index (BMI) (kg/m^2^) was calculated. Waist circumference was measured at the narrowest point between the lower costal (10th rib) border and the iliac crest. The CVs of triplicate measurements on the same day in the whole sample for height, weight, and waist circumference were 0.02%, < 0.01%, and 0.12%, respectively.

#### Pulmonary function

Resting pulmonary function tests without bronchodilators including body plethysmography and spirometry were performed^c^ at the Respiratory Function Unit, CHW. Measures included forced expiratory volume in 1 second (FEV^1^), forced vital capacity (FVC), forced mid-expiratory flow rate (FEF_25-75%_), vital capacity (VC), total lung capacity (TLC), residual volume (RV), and RV as a percentage as TLC (RV/TLC%) [49].

#### Peak aerobic capacity

The protocol treadmill test^g^ was a walking incremental incline design, set at current habitual gait speed (determined prior using an ultrasonic timer)^j^, starting at 6% grade, increasing by 2% every minute. From 24% grade, incline was maintained and speed increased by 0.5 km/hr each minute until voluntary exhaustion. Heart rate was monitored by 12-lead ECG^h^, and blood pressure was taken via an automated blood pressure system synched to ECG signal every two minutes^i^. Continuous respiratory gas analysis and volume measurements were performed breath-by-breath^k^ with a pneumotach attached to a Medgraphics mask^l^. From calorimetry data peak oxygen uptake (VO_2_peak; average of final 30 seconds of exercise) and Oxygen Uptake Efficiency Slope (OUES) [50] were calculated as measures of aerobic capacity and respiratory efficiency (OUES), where higher values are associated with better aerobic capacity.

#### Quality of life

HRQOL was measured with the Cystic Fibrosis Questionnaire-Revised (CFQ-R) [12]. Higher scores indicate better self-assessed quality of life. Questionnaires were interviewer-administered in private prior to any physical function assessments.

### Training procedure

Participants completed a four-week home-based WBV exposure, three days/week (12 sessions) with parental supervision. An investigator supervised the first session. The protocol was intermittent (1 minute vibration:1 minute rest) and progressive (Table 1). Participants stood on a Galileo Basic Platform with arms by their sides, with a 150° knee angle (slight flexion) during the exposure minute and relaxed the knee angle during the rest minute. Participants wore socks during training to prevent dampening of vibration stimulus via footwear [51], and were asked to complete a training diary and to train at the same time of day.

**Table 1:** Training Volume and Training Intensity of the Whole Body Vibration (WBV) Program

Week	Training frequency (days/week)	Vibration frequency (Hz)	Peak-to-Peak Vibration amplitude (mm)	Vibration magnitude (g)*	Total session duration (mins)	Vibration exposure (mins)
5	3	20	1	1.61	20	10
6	3	20	1	1.61	30	15
7	3	22	1	1.95	30	15
8	3	22	1	1.95	30	15

* Formula for vibration magnitude [40]: g = A (2πf)^2^/9.81, A: Peak-to-Peak Vibration amplitude (mm); f: frequency (H_z_); 9.81 = force due to gravity.

### Health status check

A researcher conducted weekly health status checks via phone to monitor acute illness, change in medications, visits to health-care professionals, new symptoms (physical, mental, emotional), bodily pain (muscular and joint), changes in mucus clearance, appetite, body mass, possible adverse effects of testing or vibration exercise and training compliance.

### Statistical analysis

Statistical analyses were performed using SPSS version 15.0^n^. The statistical approach was to use all available data regardless of compliance in this efficacy analysis, but without imputation for missing data, given the small sample size. Data distributions were inspected visually and statistically for normality (skewness -1 to +1). Normally distributed and non-normally data were described using mean ± standard deviations (SD) and median (range) respectively. Non-normally distributed data were log-transformed if possible prior to use with parametric statistics. The effect of WBV exposure was examined using repeated measures analysis of variance (ANOVA) analysis including three timepoints: baseline, pre-WBV and post-WBV. Any residual adaptation to WBV exposure retained during the washout period was examined by paired t-test using post-WBV and final timepoints. Hedge’s bias-corrected effect sizes (ES) [52-54] for the effect of WBV exposure were calculated as: Post test_mean_ - pre test_mean_/pre SD for each of the time periods. The Relative ES (= ES_intervention_ - ES_control_) [55] is reported along with 95% confidence intervals (CI). Post hoc sample size calculations were performed using calculated ES of muscle outcomes [56]. Statistical significance was accepted at P < 0.05.

## Results

### Recruitment, attrition, adverse events, and compliance

**Figure 2:**
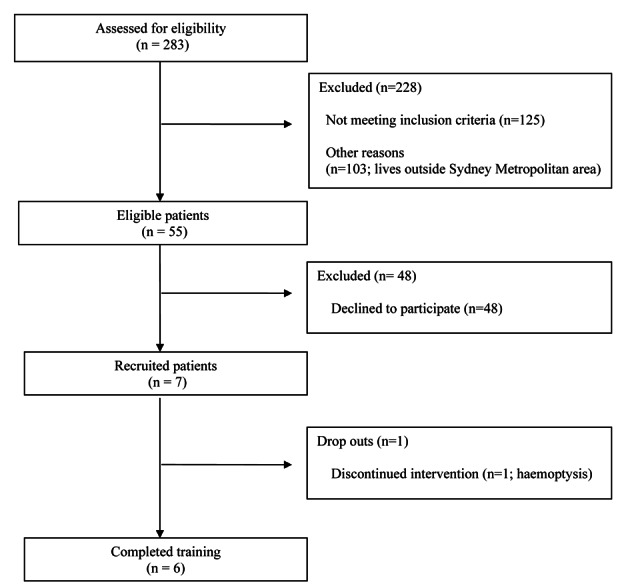
Flow diagram of participant recruitment.

From 283 patients who attend the CF clinic at CHW, 55 living in the Sydney metropolitan area fulfilled the criterion for inclusion (Fig. 2). Seven patients (four boys, three girls) were recruited, the remaining eligible subjects were not interested. One participant withdrew following one week of training after the recurrence of haemoptysis. Vibration training was not considered by medical staff to be causative. No adverse events were reported. Compliance was 100%, with participants taking, on average, 30.4 ± 1.5 days to complete the 12 sessions.

### Participant characteristics

Baseline characteristics are reported in Table 2. The mean age of participants was 11.7 ± 2.6 (range 8 - 15) years. Participants had mild to moderate CF, with only one having any hospitalisations (n = 2) in the previous 12 months. This patient subsequently withdrew from the study for an unrelated medical issue (haemoptysis). At baseline and throughout the study four participants required pancreatic enzyme supplementation. One participant suffering recurrent allergic broncho-pulmonary Aspergillosis required oral glucocorticoid medication.

**Table 2:** Participant Characteristics

Characteristic	Baseline (n = 7)
Age	11.7 ± 2.6
Sex	60% girls
Anthropometry	
Height (cm)	146.2 ± 17.7
Weight (kg)	40.8 ± 16.3
BMI (kg/m^2^)	18.3 ± 3.0
Waist circumference (cm)	67.5 ± 8.3
Health-Related Quality of Life	
Total score on CFQ-R (%) [12] (0 - 100)	83.5
Respiratory function (% of predicted)	
FEV_1_	77.4 ± 14.4
FVC	88.0 ± 16.4
VC	93.2 ± 13.6
RV	125.3 ± 92.8
TLC	99.7 ± 16.0
Aerobic Capacity	
VO_2_ peak (ml/kg/min)	34.5 ± 5.1
OUES [31]	706.4 ± 281.7
Physical Performance	
Countermovement jump height (cm)	27.0 ± 5.0
Countermovement jump power (W)	652.0 ± 306.0
Leg Extension strength (Nm)	86 ± 37
Chest Press strength (N)	213 ± 82
Leg Press strength (N)	816 ± 168
Leg Extension peak power (W)	199 ± 103
Chest Press peak power (W)	138 ± 66
Leg Press peak power (W)	473 ± 247

Values are mean ± standard deviations (SD); cm = centimetre; ml = millimetres; min = minute; kg = kilogram; cm = centimetre; m = metre; N = Newtons; Nm, Newton-metres; W = watts; % pred = value reported as percent of predicted value, 1RM = one repetition maximum; CFQ-R = cystic fibrosis questionnaire revised, where higher scores indicate better quality of life [10]; FEV_1_ = forced expiratory volume in 1 second; FVC = forced vital capacity; VC = vital capacity; RV = residual volume; TLC = total lung capacity; VO_2_peak = peak oxygen uptake during volitional maximum treadmill test; OUES = oxygen uptake efficiency slope; higher OUES is associated with higher aerobic capacity [31].

### WBV exposure

#### Main outcomes

##### 1) Muscle strength

Muscle function results are presented in Table 3. No muscle strength measures changed significantly. However, non-significant trends for improvements after WBV exposure were observed for CP strength (ES = 0.22 (-0.92, 1.35); P = 0.06), CP relative strength (ES = 0.92 (-0.27, 2.11); P = 0.10), and LE relative strength (ES = 0.66 (-0.50, 1.82); P = 0.14). It is notable that most of the trends were seen in relative measures of strength (normalised to body weight), suggesting that it was changes in neuromuscular function rather than increases in muscle mass which likely explain these effects after only four weeks of WBV exposure.

**Table 3:** Outcome Variables at Four Measurement Time Points

Outcome variable	Baseline	Pre WBV	Post WBV	Final	P value†	Effect Size 95% CI(lower, upper)	Post vs Final P value‡
Muscle Strength							
LE strength(Nm)	80.2 ± 37	80.2 ± 48.2	97.0 ± 66	92.7 ± 58.4	0.16	0.27 (-0.87, 1.41)	0.46
LE relative strength (Nm/kg)	2.0 ± 0.3	1.88 ± 0.4	2.2 ± 0.5	2.1 ± 0.4	0.14	0.66 (-0.50, 1.82)	0.51
CP strength (N)	201.0 ± 82.9	199.0 ± 86	222.3 ± 109.3	220.3 ± 104.9	0.06	0.22 (-0.92, 1.35)	0.81
CP relative strength (N/kg)	5.1 ± 0.6	4.90 ± 0.6	5.3 ± 0.3	5.3 ± 0.5	0.10	0.92 (-0.27, 2.11)	0.79
LP strength (N)	831.2 ± 197.5	838.0 ± 212.7	938.8 ± 320.4	908 ± 331.2	0.30	0.33 (-0.91, 1.58)	0.56
LP relative strength (N/kg)	21.3 ± 5.6	21.0 ± 4.9	22.9 ± 4.9	22.1 ± 5.1	0.40	0.36 (-0.89, 1.61)	0.49
Muscle Power							
LE peak power (W)	189.7 ± 108.7	196.7 ± 124.1	222.7 ± 162.2	220.3 ± 174.4	0.15	0.17 (-0.97, 1.30)	0.82
LE relative peak power (W/kg)	4.5 ± 0.8	4.5 ± 0.9	4.9 ± 1.4	5.0 ± 1.1	0.14	0.33 (-0.81, 1.47)	0.93
CP peak power (W)	132.0 ± 70.4	132.3 ± 69.1	133.3 ± 65.4	137.5 ± 74.1	0.52	0.01 (-1.12, 1.15)	0.53
CP relative peak power (W/kg)	3.2 ± 0.6	3.1 ± 0.6	3.2 ± 0.7	3.2 ± 0.5	0.97	0.06 (-1.07, 1.19)	0.98
LP peak power (W)	470.6 ± 288.7	469.2 ± 224.8	523.6 ± 251.4	519.6 ± 332	0.59	0.21 (-1.04, 1.45)	0.96
LP relative peak power (W/kg)	10.7 ± 2.1	10.7 ± 1.6	11.9 ± 1.4	9.5 ± 4.1	0.48	0.78 (-0.50, 2.07)	0.26
Counter-movement Jump (CMJ) Performance							
Height (cm)	26.3 ± 5.2	25.2 ± 4.7	27.8 ± 3.4	26.3 ± 3.3	0.11	0.60 (-0.56, 1.76)	0.17
Peak power (W)	634.2 ± 331.8	673.4 ± 375.1	721.4 ± 425.8	715.5 ± 427.3	0.13	0.11 (-1.02, 1.24)	0.77
Relative peak power (W/kg)	15.4 ± 2.2	16.1 ± 3.7	16.8 ± 2.4	167.0 ± 2.7	0.39	0.19 (-0.95, 1.32)	0.86
Peak Aerobic Capacity							
VO_2_peak (ml/kg/min)	32.3 ± 4.1	38.7 ± 5	35.6 ± 4.7	36.6 ± 2.6	0.16	-0.61 (-1.76, 0.55)	0.69
OUES	706.4 ± 281.7	953.8 ± 280.5	866.8 ± 272	840.1 ± 331.5	< 0.01	-0.28 (-1.53, 0.96)	0.62
Respiratory function							
FEV1 (%)	75 ± 14.1	80.2 ± 9.5	78.0 ± 7.5	-	0.47	-0.23 (-1.37, 0.90)	-
FVC (%)	85.5 ± 16.5	91.83 ± 11.0	89.0 ± 7.6	-	0.49	-0.28 (-1.41, 0.86)	-
FEF_25-75%_	64.7 ± 24	68.83 ± 22.2	66.0 ± 15.8	-	0.70	-0.14 (-1.27, 1.00)	-
CFQ-R	84.5 ± 7.3	83.3 ± 9.0	81.0 ± 11.5	-	0.37	-0.20 (-1.33, 0.94)	-

Values are mean ± standard deviations; Participants (WBV = 6); Effect sizes (ES) [32] were calculated as: Post testmean-pre testmean/pre SD for each of the time periods; CI = confidence interval; cm = centimetre; kg = kilogram; BMI = body mass index (kg/m^2^); Nm = Newton metres; N = Newtons; W = Watts; ml = millimetres; CP = chest press; LE = leg extension; LP = leg press;VO_2_peak = maximum oxygen uptake; OUES = oxygen uptake efficiency slope, higher values associated with higher aerobic capacity [31]; FEV_1_ = forced expiratory volume in 1 second; FVC = forced vital capacity; FEF_25-75%_ = forced mid-expiratory flow rate; Higher OUES = higher aerobic capacity; CFQ-R = Cystic Fibrosis Questionnaire-Revised, a disease-specific health-related quality of life questionnaire; higher values reflect better quality of life [10]. Normalised values are calculated as absolute strength or power/body weight in kg. *P < 0.05; † Repeated Measures ANOVA of baseline, pre, post timepoints only; ‡ paired t test to compare post and final timepoint.

##### 2) Muscle power

No muscle power outcomes changed significantly. However, among these power outcomes, LE relative power was close to significance (P = 0.14) with a small ES = 0.33 (-0.81, 1.47) and the ES for LP relative power was moderate-large (0.78 (-5.0, 2.07)). As noted above for muscle strength, it is the relative power outcomes, which tended to improve, as expected. The functional test for muscle power, CMJ height, improved by 12% compared to control after WBV exposure, and although not significant (P = 0.11) the ES was moderate ES = (0.60 (-0.56, 1.76)).

#### Secondary outcomes

There were no significant improvements after WBV exposure in pulmonary function measures or HRQOL (Table 3). Unexpectedly, OUES declined significantly after WBV exposure (P < 0.001), however VO_2_ peak did not change significantly over the course of the study.

### Washout

There were no significant differences in any outcome measures in the washout period compared to the measurement taken at the end of WBV exposure.

## Discussion

This is the first controlled trial to our knowledge assessing the effects of WBV exposure on muscle function, cardiorespiratory fitness, pulmonary function and quality of life in individuals with CF, and also the first trial ever to assess the effects of WBV exposure in children with CF. In addition, this is the first report of the effects of an extended washout period on potential adaptations to WBV. Four weeks of home-based WBV exposure had no significant effect on any primary or secondary outcome measures. However, several indices of upper and lower body relative strength and power tended to improve, with moderate-large effect sizes in some cases.

### Comparison to studies of WBV in adults with CF

The magnitude of the changes in muscle strength, power, and CMJ height we observed were similar to those reported in trials investigating WBV exposure and muscle function in adults with CF [41, 42]. Rietschel et al [41] reported that muscle power, force and velocity increased significantly after three months of training. Roth et al [42] also showed improved muscle power and velocity after 6 months of training. In Roth’s study, adults with CF improved CMJ height by 6.1% (range -25.1% to +63.9%) compared with 12% (0% to 25%) observed in our children with CF. Although these two adult studies reported significant improvements after WBV exposure, there are a number of design features that limit the robustness of these data. Most importantly, there were no control groups and both studies combined WBV exposure with lower body exercise on the platform, thus precluding attribution of benefit to WBV alone. Only one of the studies reported statistical analysis of their data [41].

### Effects on muscle strength

Ours is the first study to our knowledge to report a tendency for increased upper body strength (ES = 0.92) in patients with CF following WBV exposure. Our finding raises the possibility that WBV exposure could be beneficial to other upper body musculature such as the highly clinically relevant respiratory muscles. Children with CF rely heavily on recruitment of accessory respiratory musculature when experiencing breathing difficulties associated with pulmonary infections and progressive lung disease. Future long-term trials of WBV are needed to directly assess potential benefits to respiratory muscle function as well as rate and severity of pulmonary infections in this cohort.

### Effects on muscle power

This is the first report of study in CMJ in a clinical cohort after isolated WBV exposure. Surprisingly the improvement in CMJ height (12%) reported in our study was comparable to significant increases found after WBV with concomitant exercise in untrained females [57], young skiers [58], and healthy young adults [39]. Although none of the muscle power measures on the resistance machines changed significantly, the change in LP power, the closest measure to CMJ height, had a moderate-large ES of 0.78. The purported mechanism underlying the increase in explosive power after WBV exposure is that the vibration evokes reflex muscle fibre contractions, which in turn increases the efficiency of motor unit activation [40, 58]. It should be noted that the power measured in CMJ test allows the subject to vary both force generation as well as velocity during the movement, whereas with the pneumatic resistance machines, the load is fixed at 60% of peak strength, and the subject attempts to move it as rapidly as possible. It is possible that these differences in test requirements explain the somewhat variable effect of WBV on power outcomes in this study.

### Effects on cardiorespiratory fitness

Two prior studies in healthy adults [37, 59] have shown that cardiorespiratory fitness may improve after WBV exposure, although one of them used concomitant static and dynamic exercises on the platform, including stepping on and off it [59]. Contrary to this literature, and to our hypotheses, both aerobic capacity (P = 0.16) and OUES (P < 0.01) declined over the course of the WBV exposure. As there was no familiarisation test prior to baseline, it is likely that the apparent “gain” during the control period represents a learning effect. The average VO_2_peak at week 4 was comparable to values found in the literature for a clinical cohort of this age. For example, aerobic capacity in a cohort of 7 to 17 year old patients with CF averaged 40.2 ml/kg/min [60]. Also, as it is an effort-dependent test, standardising the performance across time-points can be difficult [15], and this may have contributed to the 7.3% decrease following WBV exposure.

### Effects on pulmonary function

Although no significant improvements in lung function were reported, this is not uncommon in exercise studies of lung diseases, as clinical benefits are generally derived from musculoskeletal rather than respiratory adaptations [1]. In-patient exercise programs in CF have failed to find any significant changes in pulmonary function following approximately two [61] or three [8] week interventions. Similarly, the two studies that have assessed WBV and exercise in adult patients with CF also reported no change to lung function following either three [41] or six [42] months of exposure. Generally, more than 12 months is required to adequately assess changes in respiratory function following exercise interventions [4, 62].

### Effect of washout period

We hypothesised that any adaptations to short term WBV exposure over four weeks would be relatively transient, as they would not be explained by changes in body composition, but likely related to acute changes in neuromuscular recruitment over repetitive bouts. This view is supported by our observation that primarily relative strength and power (normalised to body mass) exhibited trends towards improvement, precisely what one would expect if muscle mass was unchanged, but the force/power generating capacity per unit muscle was increased by WBV. Unexpectedly, however, we observed no significant difference in any outcome measures four weeks after WBV exposure ceased compared to immediately post-WBV results. This suggests that the moderate-large ES of the changes seen in relative CP and LE strength, relative LP power, and CMJ height after WBV were maintained for four weeks without additional exposure. It is possible that changes in neural recruitment patterns were somehow sustained despite withdrawal of the stimulus. It is also possible, and perhaps more likely, that repeated testing on four occasions across the study resulted in a learning/training effect on the apparati in these otherwise relatively sedentary children, such that the expected decay over the withdrawal period was masked, resulting in no change between weeks 8 and 12. We are unaware of similar time course studies after WBV withdrawal, and more information is needed on this aspect of exposure, as well as other aspects of WBV including optimal duration, frequency, intensity of g forces utilised, and dose required for maintenance.

### Study limitations

The primary limitation of the study was sample size, likely resulting in type II errors for many of our outcomes. Thus, as expected, due to the study’s intended pilot nature, our findings were not statistically significant for most outcomes. Post-hoc power calculations of the main outcomes with moderate to high ESs (Table 4) indicated that between 9 and 18 subjects in total (if control and intervention groups were separate) would be needed to demonstrate significance.

**Table 4:** Post-Hoc Power Calculations

Main outcomes	Effect Size	Total sample size required (n)
LE relative strength (Nm/kg)	0.66 (-0.50, 1.82)	10
CP relative strength (N/kg)	0.92 (-0.27, 2.11)	9
LP relative peak power (W/kg)	0.78 (-0.50,2.07)	12
Counter-movement jumpheight (cm)	0.60 (-0.56, 1.76)	18

*G-power software (GPower 3.0 for Windows, Germany) used to compute sample size necessary to achieve statistical significance assuming and two-sided alpha of 0.05 and a beta of 0.20. Sample size assumes a 2-group design, with equal 1:1 allocation to each group. Power calculations were performed for the 4 variables with the largest Effect Sizes observed in this pilot study.

We acknowledge other limitations to our design. There was no blinded outcome assessor. Treatment order was not randomised, as we could not exclude the possibility of a carry-over effect. A lack of familiarisation tests could have resulted in learning effects at follow-up which could have masked decay after withdrawal. We had no direct measures of body composition, muscle metabolism, or neural activation. Four weeks of WBV exposure may not have been sufficient to maximally stimulate musculoskeletal adaptations. The optimal dose of WBV for muscle function has not been determined in this population in terms of g forces, intermittent vs. continuous exposure, duration and/or frequency of exposure. Positioning of the knees while standing, placement of hands on a support rail connected to the vibrating platform, timing relative to bedtimes, meals, treatments or other activities, and use of concomitant exercises are all variables that require further study.

### Conclusions and future directions

Our results suggest the potential for WBV exposure to improve both upper and lower body strength and power, although the findings are clearly preliminary and in need of replication and expansion in appropriately powered studies. With the life expectancy of the CF population increasing, sarcopenia and associated muscle dysfunction related to the disease and pharmacologic therapy with glucocorticoids will become an increasingly important clinical problem, WBV should also be examined in older patients and those with more severe lung dysfunction, as they are even more likely to be inactive than children with CF and have greater exercise intolerance and co-morbidity, and thus may potentially benefit more from the low exertion nature of WBV. A training program of longer duration should be investigated, with testing performed periodically over the study duration to monitor rates of change. With longer intervention duration, changes in muscle size and morphology should be assessed, to examine whether WBV can induce hypertrophy, as reported in one [63], but not another [64], long-term study. Given the large effect size we observed in upper body relative strength changes, future WBV research should include more measures of upper body muscle function including respiratory muscle function, to investigate the reproducibility and clinical utility of these findings. Dose-response characteristics and detraining effects remain to be clarified, as well as clinical benefits and changes in quality of life for this vulnerable cohort.

## Suppliers

^a^Keiser Sports Health Equipment, Inc., Fresno, CA. ^b^Kistler 9281B, Wintertur, Switzerland. ^c^Sensormedics 2000; Sensormedics, Yorba Linda, CA. ^d^Stadiometer; Holtain Limited, Crymmych Pembs., UK. ^e^Electronic scale; HW-100K, A&D Bench Scales, Bradford, MA. ^f^Lufkin steel tape measure, W606 PM. ^g^Quinton Q-Stress TM55 Treadmill, Quinton Cardiology Systems, Inc; Bothell, WA, U.S.A. ^h^Quinton 4500 Stress Test Monitor, Quinton Cardiology Systems, Inc; Bothell, WA, U.S.A. ^i^Quinton Automatic BP monitor model 412; Bothell, WA, U.S.A. ^j^Ultratimer, DCPB Electronics; Glasgow, Scotland. ^k^Ultima PFX Series, Medgraphics; St. Paul, Minnesota, U.S.A. ^l^Pat. #6718982, Taiwan. ^m^Novotech Medical, Pforzheim, Germany. ^n^Version 15.0 for Windows,SPSS Inc. Chicago, IL. ^o^GPower 3.0 for Windows, Germany. http://www.psycho.uni-duesseldorf.de/abteilungen/aap/gpower3
